# Misdiagnosed psychiatric manifestations in a rare disease: a case report of secondary anxiety syndrome in Cushing’s disease

**DOI:** 10.3389/fpsyt.2023.1190899

**Published:** 2023-04-27

**Authors:** Wenqi Geng, Lijia Cui, Tao Li, Xueqing Liu, Yong Yao, Xia Hong, Huijuan Zhu, Lin Lu, Jing Wei

**Affiliations:** ^1^Department of Psychological Medicine, Peking Union Medical College Hospital, Chinese Academy of Medical Sciences and Peking Union Medical College, Beijing, China; ^2^Department of Endocrinology, Key Laboratory of Endocrinology of National Health Commission, Peking Union Medical College Hospital, Chinese Academy of Medical Sciences and Peking Union Medical College, Beijing, China; ^3^Department of Radiology, Peking Union Medical College Hospital, Chinese Academy of Medical Sciences and Peking Union Medical College, Beijing, China; ^4^Department of Neurosurgery, Center for Pituitary Surgery, China Pituitary Disease Registry Center, China Pituitary Adenoma Specialist Council, Peking Union Medical College Hospital, Chinese Academy of Medical Sciences and Peking Union Medical College, Beijing, China

**Keywords:** anxiety disorders, Cushing syndrome, pituitary adenoma, serotonin, referral and consultation

## Abstract

Diagnosing and treating secondary psychiatric symptoms with accuracy can be challenging in clinical settings. In this case study, we report on a female patient with Cushing’s disease who was misdiagnosed with anxiety disorder during her first psychiatric visit. Following initial ineffective psychiatric intervention, unexplained hypokalemia, and hypothyroidism, the patient visited the endocrinology clinic and was diagnosed with Cushing’s disease. During the medical and surgical procedures that followed, high doses of psychotropic medication were continued to treat persisting anxiety. After discharge, the patient developed autonomic dysfunction and impaired consciousness. Upon readmission, serotonin syndrome due to inappropriate psychiatric medication was diagnosed. The management of secondary psychiatric syndromes must be adapted to changes in the patient’s primary condition, which necessitates interdisciplinary collaboration in general hospital settings.

## 1. Introduction

Managing secondary psychiatric symptoms can be challenging, especially for rare medical conditions with little experience in therapy. Cushing’s syndrome (CS), also known as hypercortisolism, is characterized by hypercortisolemia with various causes. As a rare condition, its incidence is estimated to be 2–3 per million per year (1, 2). The term Cushing’s disease (CD) refers to CS caused by pituitary adrenocorticotropic hormone (ACTH) adenomas. Hypercortisolism predisposes patients to psychopathology, mainly depression and anxiety symptoms (3). After cortisol normalization leading to remission of CS, improvements of previous psychiatric alterations may occur to some extent gradually over time, although there is controversy as to complete recovery (4). To our knowledge, there have been a few reports on the trajectories of anxiety symptoms after recovery from CS (5–7), but no specific experience in treating anxiety in patients with CS have been shared. Herein, we report a case of CD that was initially misdiagnosed with primary anxiety disorder and major depressive episode, and which developed serotonin syndrome due to psychiatric medication after surgical removal of ACTH adenoma.

## 2. Case presentation

### 2.1. Outpatient visits and hospitalization in psychiatric facility

**Figure 1:**
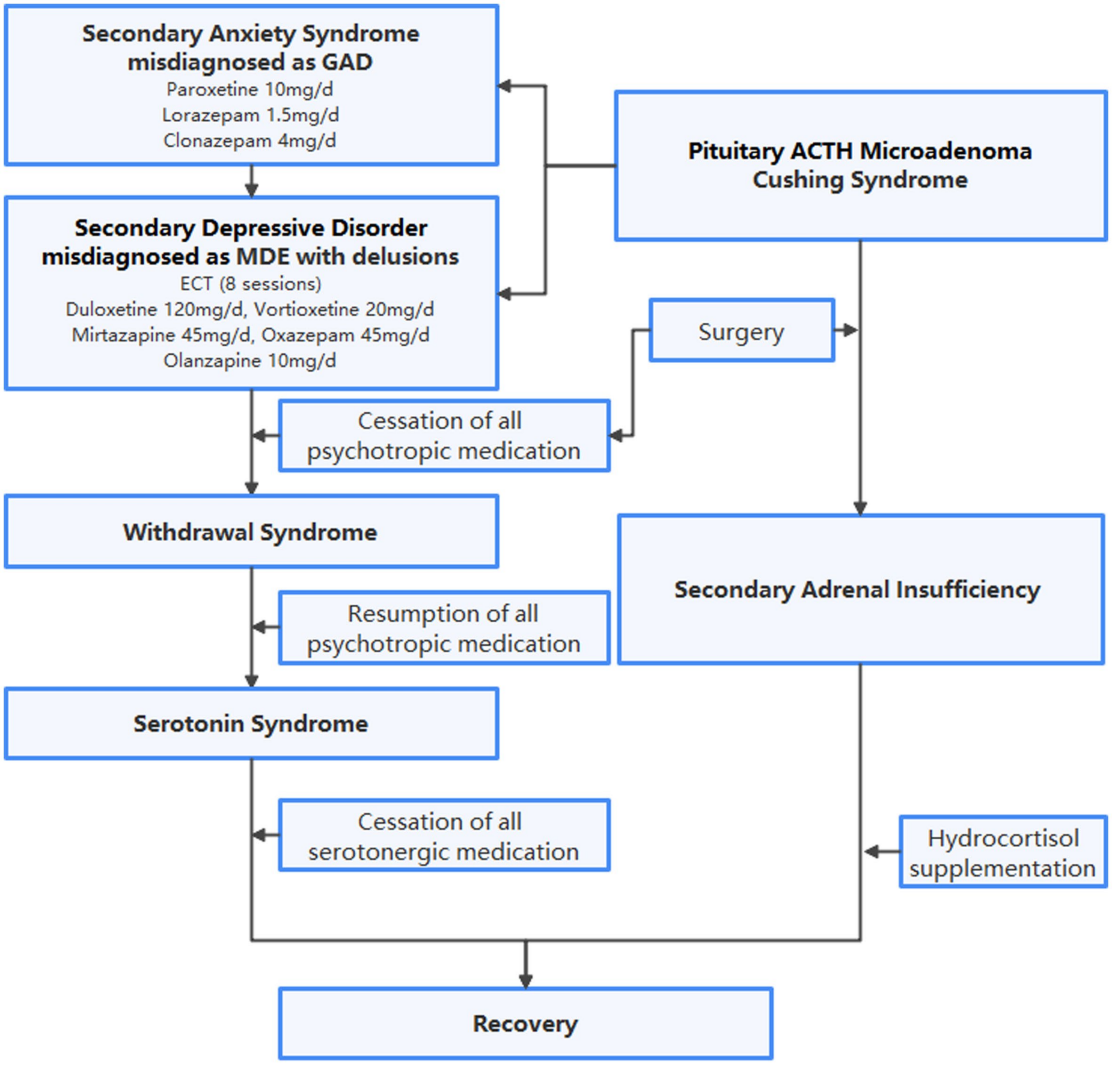
Flowchart of diagnoses and treatment. GAD, generalized anxiety disorder; MDE, major depressive episode; ECT, electroconvulsive therapy; ACTH, adrenocorticotropic hormone.

A 47-year-old woman, accompanied by her husband, voluntarily visited the psychiatric clinic after 2 months of anxiety, weight loss and sleep problems linked to work stress. The patient complained of constant nervousness, muscular tension, restlessness, sweating, heart palpitations, dizziness, and epigastric discomfort. She has been exhibiting obsessive behavior since adolescence. The patient has no family history of mental disorders or severe physical diseases. Laboratory tests, including complete hemogram, electrolytes (sodium, potassium, calcium, magnesium, chlorine), and thyroid function, were all within normal range. For a preliminary diagnosis of anxiety disorder, unspecified, paroxetine 20 mg/d and lorazepam 1.5 mg/d were prescribed. Lorazepam was changed to clonazepam 4 mg/d after one week due to unresolved anxiety, restlessness, and insomnia. Following a panic attack, the patient visited the emergency department, which revealed hypokalemia (potassium 3.0 mmol/l). She gradually developed depression and delusions as more symptoms appeared, convinced that all physical symptoms were signs of severe organ dysfunction, which led her to have suicidal thoughts. Following a suicide attempt, she was involuntarily admitted to another psychiatric facility. During hospitalization, blood tests revealed persistent hypokalemia and progressive hypothyroidism, which could not be fully explained by inadequate dietary intake. Immunologic markers, including autoantibodies, immunoglobins, and complements, were not remarkable. A CT scan of the head yielded no significant findings. Electroconvulsive therapy and a variety of medications were utilized to treat major depressive episode with nihilistic delusion, as shown in Figure 1. Upon discharge, the patient achieved clinical remission of depression. Nevertheless, anxiety was still present, and an undiscovered physical condition was suspected.

### 2.2. Discovery and surgery of pituitary microadenoma

**Figure 2:**
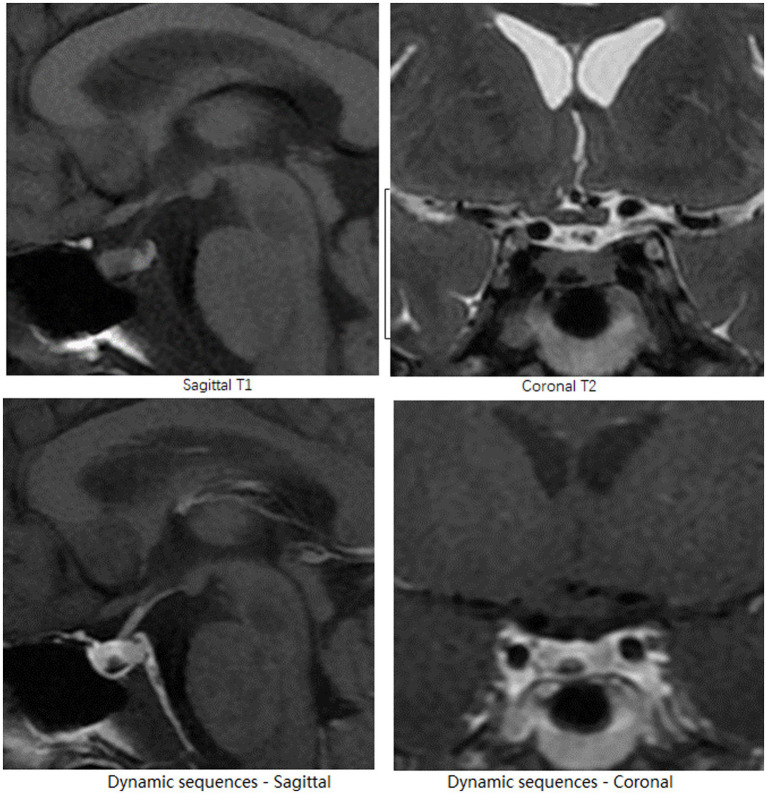
Head MRI. On the right side of the pituitary gland, the pituitary microadenoma, appears as a nodular lesion with a hypointense signal on sagittal T1-and coronal T2-weighted pre-enhanced MRI images, and demonstrates delayed hyperenhancement on dynamic sequences.

After presenting to the Department of Endocrinology, the patient underwent an enhanced MRI of the head and found a microadenoma 0.3 cm in diameter to the right side of the lower pituitary gland, as shown in Figure 2. In a further review of symptoms, the patient recalled a rounder face and increased waist circumference prior to anxiety symptoms, and amenorrhea following anxiety symptoms and reduced food intake. Purple striae and buffalo hump were absent, as were signs of secondary diabetes, hypertension, or osteoporosis. Laboratory tests revealed elevated serum free cortisol, ACTH, and 24-h urinary free cortisol (24-h UFC), and the phenomenon that 24-h UFC and serum cortisol was not suppressed after low-dose dexamethasone suppression test, all indicated the diagnosis of CS. Elevated ACTH suggested the possibility of ACTH-dependent CS. Furthermore, bilateral petrosal sinus sampling (BIPSS) with desmopressin stimulation test showed inferior petrosal sinus/peripheral (IPS/P) ACTH gradient at baseline as 3.1, and IPS/P ACTH gradient after desmopressin stimulation as 10.6. Taking all of the above into account, the patient was diagnosed with CD (8) and subsequently transferred to the Department of Neurosurgery for pituitary microadenoma resection. Psychiatric medication was not discontinued or reduced until the day of surgery. The day after surgery, the patient developed tachycardia, tachypnea, elevated temperatures, tremors, and strong anxiety. Over the next few days, her condition progressed to hypovolemic shock. She refused to take oral medication and refused to cooperate with verbal responses. Considering that the above-mentioned manifestations are due to postoperative adrenal insufficiency, hydrocortisone supplementation was introduced. All psychiatric medications were resumed to alleviate anxiety. After resumption of medication, extrapyramidal symptoms were observed during psychiatric consultations, which prompted recommendations for tapering psychotropic agents. Before discharge, the patient maintained the preoperative dose of all antidepressants, anxiolytics, and antipsychotics because her anxiety had not improved.

### 2.3. Readmission with severe medical condition

**Figure 3:**
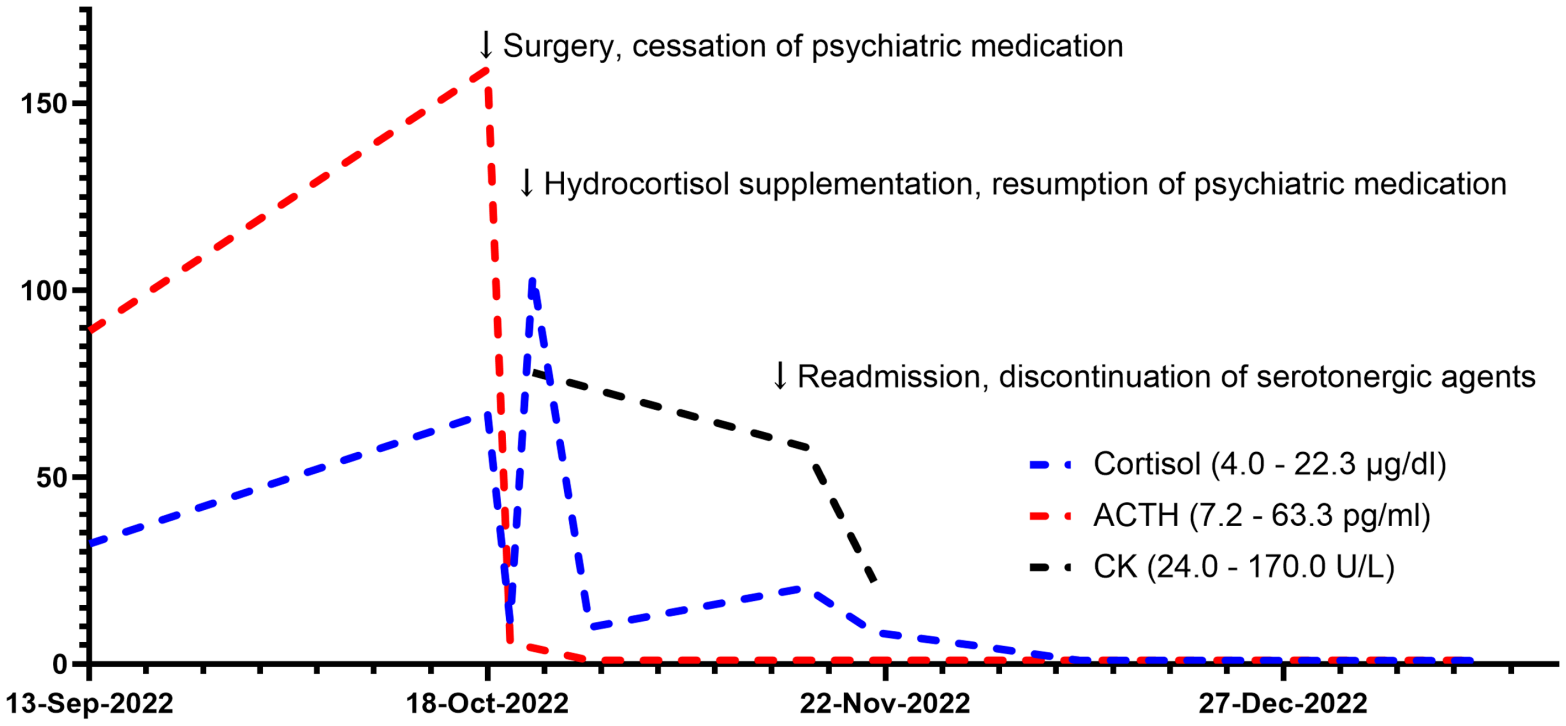
Trajectories of cortisol and ACTH. ACTH, adrenocorticotropic hormone. CK, creatine kinase.

One week after discharge, the patient was again admitted to the emergency department with a high fever, tachycardia, hypertension, and impaired consciousness. The initial diagnosis was secondary adrenal insufficiency after excluding infection. Physical examination indicated tremor, dysarthria, increased muscle tone in the extremities and neck, and myoclonic manifestations of the lower extremities, all of which could not be fully explained by adrenal insufficiency. A psychiatric follow-up consultation revealed that the patient was still taking multiple antidepressants due to persistent anxiety, raising the possibility of serotonin syndrome. Serotoninergic medications were recommended to be discontinued despite the discovery of normal creatine kinase level, with only clonazepam remaining to treat anxiety. Blood pressure, heart rate, and temperature all decreased to normal the next day, as did muscle spasticity. The patient’s subjective anxiety slowly subsided by the time she was discharged 4 weeks later. Clonazepam was gradually replaced by estazolam due to daytime sleepiness. The trajectories of serum cortisol, ACTH, and creatine kinase are shown in Figure 3. During the one-month follow-up after discharge, the patient expressed concern for her health and made plans to eventually resume family and work responsibilities once her condition stabilized.

## 3. Discussion

This case report describes the management of psychiatric symptoms of a patient with Cushing**’**s disease. Symptomatic treatment based on improvement of the primary condition is the guiding principle for the treatment of secondary psychiatric syndromes. Managing psychiatric symptoms can be challenging, however, for rare medical conditions with little experience in therapy.

### 3.1. Anxiety throughout the illness course

Although the patient has experienced a depressive episode with delusional symptoms, anxiety is the psychiatric manifestation that persists throughout the course of the illness. With the hypothalamo-pituitary–adrenal (HPA) axis playing a crucial role, anxiety and stress responses are closely related (9, 10). The high prevalence of psychiatric symptoms in CD may be explained by structural and functional changes in the central nervous system, including the hippocampus (11) and amygdala (12, 13), caused by chronic hypercortisolism and HPA axis dysfunction (4). In addition, CD, as a chronic disease, can also cause serious dysfunction and impair quality of life, resulting in adaptation difficulties that frequently result in the development of anxiety and depressive symptoms (14). Since the patient’s insidious physical symptoms predated the onset of anxiety symptoms in this case but did not result in significant psychosocial stress, we assume that anxiety is more neurobiological in nature.

The possibility of some gradual improvement in previous psychiatric alterations over time after cortisol normalization results in CS remission is debatable (4). Some studies suggest that anxiety symptoms tend to improve over time, although subclinical or trait anxiety may remain (6, 7, 15). Secondary adrenal insufficiency is another point to take into account. It has been demonstrated that primary adrenal insufficiency can manifest a variety of possible psychiatric symptoms, such as psychosis, depression, and anxiety (16). In addition, there have been reports of psychosis and delirium in secondary adrenal insufficiency as a result of opiate replacement medication (17), radiotherapy of pituitary adenoma (18), and empty sella syndrome (19). In this case, the patient’s continued concern for her health after discharge may be explained by psychosocial reactions associated with disease adaptation. Despite this, secondary adrenal insufficiency cannot be completely ruled out.

### 3.2. Psychotropic medications for secondary psychiatric syndromes

Endocrine disorders are a frequent cause of secondary anxiety syndrome (20). Persistence of affective disorders associated with prior hypercortisolism can have a sustained impact on well-being and quality of life in patients (21, 22). Psychiatric medications, such as antidepressants, anxiolytics, and antipsychotics, are typically viewed as treatments that alleviate symptoms and are prescribed both prior to and after diagnosis (21, 23). In this case, the patient’s initial symptoms of anxiety were unresponsive to regular treatment. One anxiolytic and one antipsychotic were added to three full-dose antidepressants, and anxiety persisted until additional abnormal blood test results were found. Subsequently, the patient underwent a rollercoaster of dose adjustments before and after surgery. Withdrawal syndrome frequently results from abrupt discontinuation of high doses of antidepressants, antipsychotics, and anxiolytics (24). Sudden cessation of antidepressants will result in a sharp drop in serotonin concentration, which will increase anxiety and depressive symptoms (25). We speculate that cortisol’s aggravation of anxiety symptoms gradually diminishes after pituitary adenoma has been surgically removed. Resuming the same dose of antidepressants results in the development of serotonin syndrome because the actual serotonin level is significantly higher than what is required. Meanwhile, the combination of the three antidepressants itself predisposes the patient to serotonin syndrome. Clinical differential diagnosis is extremely challenging because both serotonin syndrome and secondary adrenal insufficiency can lead to hyperthermia, autonomic dysfunction, and impaired consciousness (16, 18). Nevertheless, we made the decision to discontinue serotonergic medications and only retain benzodiazepines in this case, as the patient’s presentation clearly matched serotonin syndrome. Another evidence to support serotonin syndrome versus secondary adrenal insufficiency was that at the occurrence of hyperthermia, autonomic dysfunction, and impaired consciousness, the patient was still taking hydrocortisone 60 mg/d which is beyond the physiological dose. The patient’s subsequent improvement was, in fact, consistent with the characteristics of rapid remission of serotonin syndrome (26, 27). It is essential to regularly evaluate symptoms of secondary psychiatric syndromes alongside the improvement of the primary condition, while psychiatric medications must be modified accordingly to reduce adverse effects and prevent serious consequences.

In conclusion, organic anxiety should be taken into account when the conventional anxiolytic regimen does not respond well. It is not advisable to combine multiple full-dose antidepressants, which significantly increases the risk of developing serotonin syndrome. The management of secondary psychiatric symptoms must be adapted to changes in the patient’s primary condition. To support optimal outcomes in secondary psychiatric syndromes, multidisciplinary collaboration and routine follow-up should be emphasized.

## Data availability statement

The raw data supporting the conclusions of this article will be made available by the authors, without undue reservation.

## Ethics statement

The studies involving human participants were reviewed and approved by The Ethics Committee of Peking Union Medical College Hospital. The patients/participants provided their written informed consent to participate in this study. Written informed consent was obtained from the individual(s) for the publication of any potentially identifiable images or data included in this article.

## Author contributions

WG, XH, and JW: conceptualization. WG, TL, and XL: data curation. WG: writing–original draft. WG, XH, and LC: writing–review and editing. JW, YY, HZ, and LL: supervision. All authors contributed to the article and approved the submitted version.

## Funding

This work was supported by the Capital Funds for Health Improvement and Research (grant number: CFH 2022-2-4012) and the STI2030-Major Projects (grant number: 2021ZD0202001). Funders played no role in the content of this article.

## Conflict of interest

The authors declare that the research was conducted in the absence of any commercial or financial relationships that could be construed as a potential conflict of interest.

## Publisher’s note

All claims expressed in this article are solely those of the authors and do not necessarily represent those of their affiliated organizations, or those of the publisher, the editors and the reviewers. Any product that may be evaluated in this article, or claim that may be made by its manufacturer, is not guaranteed or endorsed by the publisher.
